# Synthesis of 2-Phenylazonaphtho[1,8-*ef*][1,4]diazepines and 9-(3-Arylhydrazono)pyrrolo[1,2-*a*]perimidines as Antitumor Agents

**DOI:** 10.3390/molecules19010740

**Published:** 2014-01-08

**Authors:** Thoraya A. Farghaly, Eman M. H. Abbas, Kamal M. Dawood, Tarek B. A. El-Naggar

**Affiliations:** 1 Department of Chemistry, Faculty of Science, University of Cairo, Giza 12613, Egypt; E-Mail: dr_dawood@yahoo.com; 2 Department of Chemistry, Natural and Microbial Products, National Research Center, Dokki, Cairo 12622, Egypt; E-Mails: eman_m69@yahoo.com (E.M.H.A.); telnaggar@yahoo.com (T.B.A.E.-N.); 3 Departamento de Farmacología, Facultad de Farmacia, Universidad Complutense de Madrid, Plaza de Ramón y Cajal s/n, Madrid 28040, Spain

**Keywords:** 1,8-diaminonaphthalene, naphtho[1,8-*ef*][1,4]diazepines, pyrrolo[1,2-*a*] perimidines, hydrazonoyl chloride, antitumor activity

## Abstract

Two series of naphtho[1,8-*ef*][1,4]diazepines and pyrrolo[1,2-*a*]perimidines were prepared starting from 1,8-diaminonaphthalene and hydrazonoyl chlorides. The structures of the products were determined on the basis of their spectral data and elemental analyses. The mechanism of formation of such products was also discussed. The prepared compounds were screened for their antitumor activity against three cell lines, namely, MCF-7, TK-10 and UACC-62, and some derivatives showed promising activity.

## 1. Introduction

Naphthalene is important aryl ring found in many biologically active compounds such as anti-inflammatory [1], anti-bacterial [2], anti-microbial [3] and anti-cancer agents [4]. 1,8-Diaminonaphthalene was used in synthesis of a limited number of naphtho[1,8-*ef*][1,4]diazepine derivatives [5,6,7,8] and many perimidine derivatives [9,10,11,12]. Perimidines are an important class of heterocyclic systems due to their biological and pharmaceutical activities [9,10]. They have been used as anti-ulcer, anti-microbial, and anti-fungal agents [11,12]. Perimidines have also been described as DNA-intercalating and anti-tumor agents against several carcinogenic cell lines [13,14,15,16]. Very recently, we reported some perimidine anti-tumor agents that showed good activity against the MCF-7 breast cancer cell line and HEPG-2 liver cancer cell line [17]. In addition, perimidines constitute the core of several dyes [18], and are used in the synthesis of photovoltaic devices [19] and as a source of carbene ligands [20]. Furthermore, pyrrolo[1,2-a] perimidines are an interesting class of fused heterocycles that have wide range of industrial applications as dyes and pigments for plastics [21,22,23,24]. From the above findings and in continuation of our research work on the use of hydrazonoyl halides in the synthesis of biologically active heterocycles [25,26,27,28,29,30,31,32], we report here the so far unreported reaction between 1,8-diaminonaphthalene and hydrazonoyl chlorides as a key step for synthesis of the naphtho[1,8-*ef*][1,4]diazepine derivatives. We also synthesized a new perimidine derivative to study its regioselective reaction towards a number of hydrazonoyl chlorides. The effects of the newly synthesized compounds on the growth of three human cancer cell lines: MCF-7, TK-10, UACC-62, are also examined.

## 2. Results and Discussion

### 2.1. Chemistry

Treatment of 1,8-diaminonaphthalene (**1**) with the acetylhydrazonoyl chloride **2a** in dioxane in the presence of triethylamine at reflux temperature furnished a single product to which the structure 2-(4-methoxyphenylhydrazono)-3-methyl-1*H*-naphtho[1,8-*ef*] [1,4] diazepine (**3a**) was assigned based on the elemental analyses and spectral data of the isolated product. The IR spectrum of the reaction product showed two NH absorption bands at 3,394 and 3,286 cm^−1^. Moreover, the mass spectrum of the isolated product revealed a molecular ion peak at *m/z* 330. ^1^H-NMR displayed two singlet peaks resonating at 2.25 and 3.58 due to the CH_3_ and OCH_3_ protons, respectively, in addition to two broad D_2_O-exchangeable signals resonating at 9.98 and 10.10 due to two NH protons, in addition to an aromatic multiplet in the 6.65–7.92 ppm region.

**Scheme 1:**
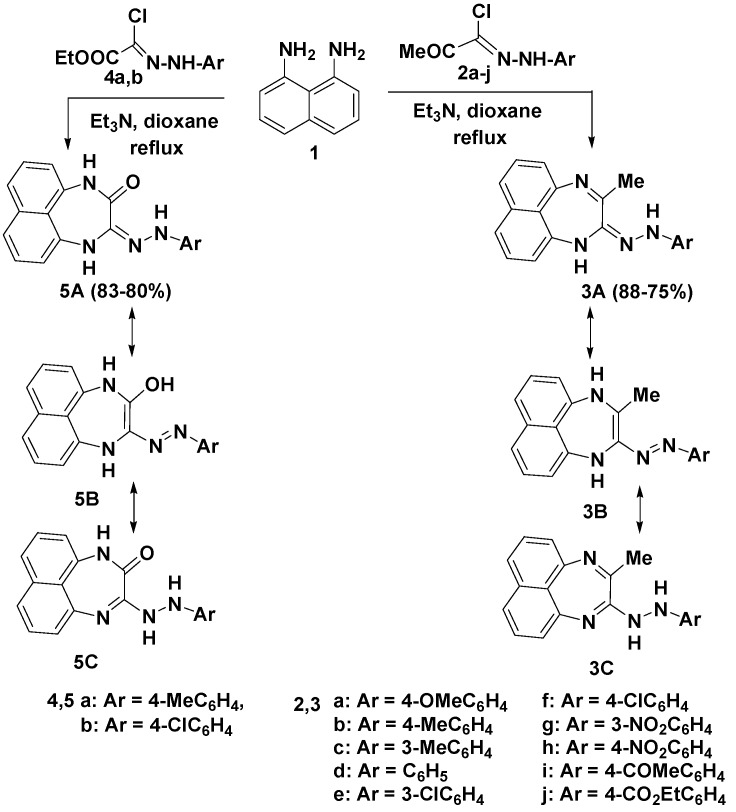
Reaction of 1,8-diaminonaphthalene (**1**) with hydrazonoyl chlorides **2** and **4**.

Analogous reactions of the acetyl hydrazonoyl chlorides **2b**–**j** with 1,8-diaminonaphthalene (**1**) furnished the corresponding 2-(arylhydrazono)-3-methyl-1*H*-naphtho[1,8-*ef*][1,4] diazepine derivatives **3b**–**j** in high yields, as outlined in Scheme 1. The structures of the isolated products were assigned on the basis of their elemental analyses and spectral data (see Experimental section). Compounds **3** have three tautomeric forms **3A**–**C** (Scheme 1). The electronic absorption spectra of **3 **in dioxane revealed, in each case, one characteristic absorption band in the region 381–345 nm (Table 1) and the electronic absorption spectra of compound **3d**, taken in different solvents as a typical example of the series prepared, exhibited little, if any, solvent dependence (Table 1). These data indicate that compound **3** is found mainly in the hydrazone-form **3A** [33,34].

Similar reaction of 1,8-diaminonaphthalene (**1**) with the ester hydrazonoyl chlorides **4a**,**b**, under the typical experimental reaction conditions mentioned above, led to the formation of the corresponding 2-(arylhydrazono)-1,4-dihydro-naphtho[1,8-*ef*][1,4]diazepine-3-ones **5a**,**b**. The structure elucidation of the reaction products **5a**,**b** was based on their elemental analyses and spectral data (IR, MS and ^1^H-NMR). The IR spectra of the reaction products exhibited three NH absorptions in the 3,325–3,120 cm^−1^ region in addition to an amide -C=O absorption band around 1,660 cm^−1^, as well as three singlet D_2_O-exchangeable-NH proton signals resonating in the 9.95–10.91 region in their ^1^H-NMR spectra. Moreover, their mass spectra revealed in each case a peak due to the molecular ion (M^+^)*.* The electronic absorption spectra data of **5** in dioxane revealed, in each case, one characteristic absorption band in the 378 and 385 nm region corresponding to the hydrazone chromophore **5A** [33,34] (Table 1).

**Table 1:** UV Spectral data of compounds **3a**–**j**, **5a**,**b** and **11a**–**h** in dioxane.

Compd. No.	λ_max_ (log ε)	Compd. No.	λ_max_ (log ε)
**3a**	359 (4.25)	**5a**	378 (4.45)
**3b**	349 (3.89)	**5b**	385 (4.33)
**3c**	347 (3.59)	**11a**	484 (4.24), 346 (4.19), 269 (4.24)
**3d** *	348 (4.30)	**11b**	476 (4.01), 340 (4.10), 233 (4.28)
**3e**	349 (3.87)	**11c** **	473 (4.15), 351 (4.32), 231 (4.38)
**3f**	349 (3.88)	**11d**	472 (4.24), 339 (4.15), 232 (4.35)
**3g**	345 (3.89)	**11e**	472 (4.0), 337 (3.89), 267 (4.01)
**3h**	381 (3.76)	**11f**	490 (4.66), 357 (4.57), 231 (4.89)
**3i**	359 (4.19)	**1g**	509 (4.05), 361 (4.11), 262 (4.13)
**3j**	351 (4.05)	**11h**	478 (4.10), 336 (3.79), 296 (3.80)

* Solvent λ_max_ (log ε): acetone: 349 (4.01); chloroform: 348 (4.02); cyclohexane: 347 (4.11); ethanol 348 (4.04); DMF 349 (4.24); ** Solvent λ_max_ (log ε): acetone: 473 (4.20), 350 (4.35), 231 (4.41); chloroform: 473 (3.99), 353 (4.15), 233 (4.28); cyclohexane: 473 (4.25), 350 (4.10), 230 (4.08); ethanol 472 (4.24), 352 (4.23), 236 (4.47); DMF 476 (4.36), 346 (4.29), 231 (4.38).

**Scheme 2:**
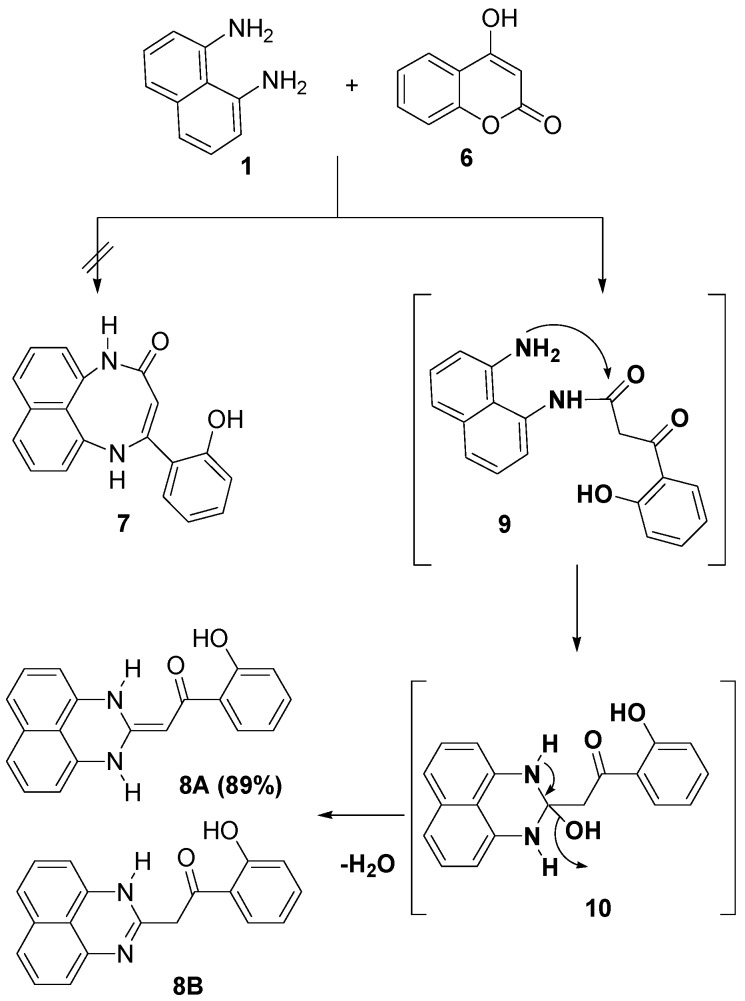
Reaction of 1,8-diaminonaphthalene (**1**) with 4-hydroxycoumarin (**6**).

From the literature [35,36], we found that reaction of 4-hydroxycoumarin with 1,2-phenylenediamine furnished 1,5-benzodiazepin-2-ones. This finding attracted our intention to study the reaction of 4-hydroxycoumarin with 1,8-diaminonaphthalene. Thus, heating a 1:1 molar mixture of 1,8-diaminonaphthalene (**1**) with 4-hydroxycoumarin (**6**) in refluxing ethanol furnished a single product for which two possible structures; the naphtho[1,8-*bc*][1,5]diazocin-2-one structure **7** or the 1,2-dihydroperimidine **8** derivatives can be postulated (Scheme 2). Based on its spectral data (MS, IR, ^1^H-NMR and ^13^C-NMR) the 2-(2-hydroxybenzoylmethylene)-1,2-dihydroperimidine structure **8** was assigned to the reaction product. For example, its ^13^C-NMR revealed a characteristic signal at δ 186.22 due to a ketonic carbonyl-carbon rather than an amidic carbonyl-carbon where the latter one resonates in the region δ 160–166 [25] and the signal for methene carbon at 77.15 [37]. Also, the carbon signals of the perimidine moiety were clearly observed in the ^13^C-NMR spectrum of compound **8** [37]. Further evidence for the assigned structure **8** is provided by its mass spectrum, which revealed ion peaks at *m/z* 168 and 121, corresponding to the perimidine and HO-C_6_H_4_-CO fragments, respectively. This assignment is in good agreement with literature data indicating the stability of perimidines to be greater than that of diazocines [37,38,39].

To account for the formation of the product **8**, it is suggested, as depicted in Scheme 2, that the reaction of **1** with **6** starts with two nucleophilic addition reactions to give the intermediates **9** and **10** which underwent *in situ* cyclization via elimination of water molecules to give **8** as the end product. Compound **8** has two possible tautomeric forms, **8A** and **8B**, however the presence of a singlet signal resonating at 5.62 integrated for 1 H due to (-C=CH-) and the absence of any aliphatic protons in the 2–4 region of the ^1^H-NMR confirmed the tautomer **8A** as the main product (Scheme 2).

**Scheme 3:**
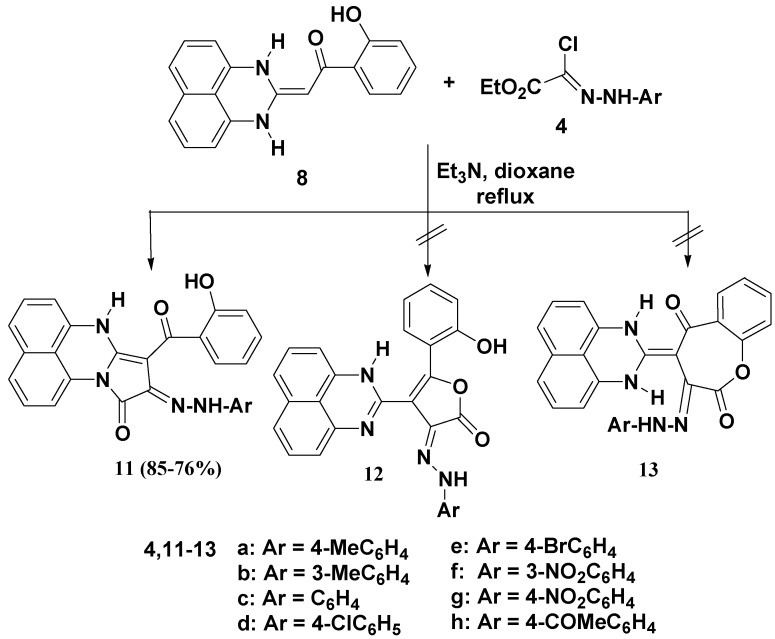
Reaction of compound **8** with hydrazonoyl chlorides **4a**–**h**.

The reactivity of the multifunctional -enaminoketone **8A** towards the hydrazonoyl chlorides **4** was also investigated. Both the -enaminoketone **8A** and hydrazonoyl chlorides **4** have reaction different sites that may attack each other, thus, when compound **8A** was treated with the ester hydrazonoyl chlorides **4a**–**h** in refluxing dioxane in the presence of triethylamine, it afforded, in each case, only one isolable product whose elemental analyses and mass spectral data were consistent with compounds **11**, **12** or **13** (Scheme 3), where all products have identical molecular ion peaks due to elimination of HCl and ethanol molecules.

**Scheme 4:**
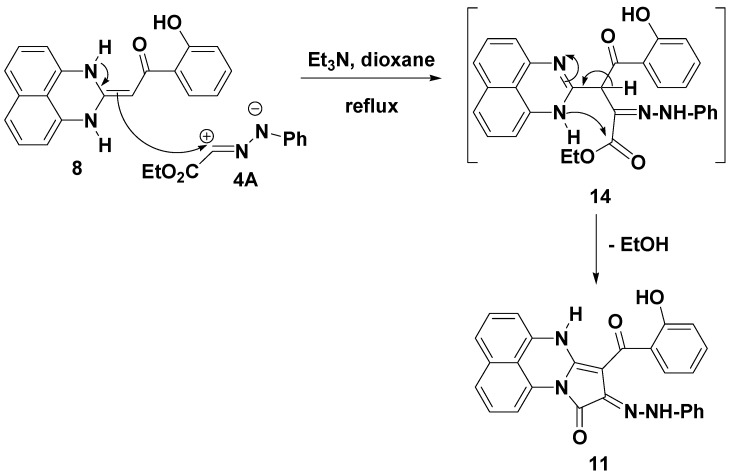
The mechanism of formation of compounds **11**.

**Figure 1:**
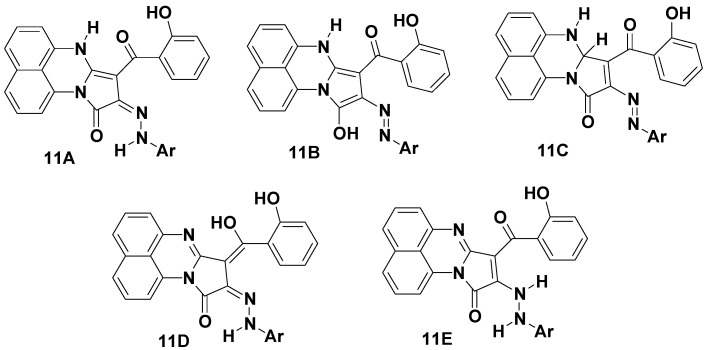
Tautomeric structures of compounds **11**.

The IR spectra of the products in the solid state showed carbonyl group bands in the 1693–1687 cm^−1^ (cyclic amide C=O group) and 1647–1639 cm^−1^ (benzoyl C=O group) regions, which are consistent with the structure of compound **11** rather than its isomeric structures **12** or **13** (Scheme 3). The mechanism outlined in Scheme 4 accounts for the formation of compound **11**. Due to the conjugation effect of the electron-donating amino group of compound **4** and the electron-withdrawing substituent (2-hydroxybenzoyl group), the double bond is highly polarized and the electron density on the α-carbon is greater than that of the nitrogen atom so the reaction is assumed to proceed through carbon-carbon cross-coupling via initial attack of the activated -enamine’s carbon (nucleophile) of **8A** on the electrophilic carbon of nitrilimine **4A**, followed by elimination of ethanol to give the desired product **11** as depicted in Scheme 4. As products **11** can have five possible tautomeric structures **11A**–**E**, their electronic absorption spectra were studied in dioxane and showed in each case three absorption bands in the 509–472, 361–336 and 296–231 nm regions (Figure 1). This absorption pattern is similar to that reported for analogous azo chromophore [40]. Such data indicate that the actual tautomeric structure of such compounds in solution phase is the arylazohydroxy form **11B**, whereas their IR spectral data indicate that they exist in the solid state as the diketo-hydrazone tautomeric form **11A** (Figure 1).

### 2.2. Results of Biological Activity

#### 2.2.1. Effect on the Growth of Human Cancer Cell Lines

The effects of the 16 newly synthesized compounds **3c**–**f**, **3h**–**j**, **8**, **11a**–**h** on the growth of three human cancer cell lines, TK-10, MCF-7 and UACC-62, given in concentrations that were able to cause 50% of cell growth inhibition, are summarized in Table 2. Regarding the activities of the naphtha[1,8-*ef*]diazepine derivatives **3**, no capacity to inhibit the growth of the human cancer cell lines was observed with **3e** and **3h**, while compound **3i** inhibited the GI_50_ on all the cell lines (TK-10 = 96.73 µM/mL, MCF-7 = 81.29 µM/mL and UACC-62 = 41.97 µM/mL). Compounds **3c** and **3j **showed an inhibitory effect against the growth of the TK-10 and MCF-7 cells, but did not possess any activity on the UACC-62 cell line. Compounds **3d** and **3f** showed an inhibitory effect against one cell line only (MCF-7 and TK-10 cells, respectively). On the other hand, regarding the activities of the pyrrolo[1,2- α]perimidine derivatives **11a**–**h**, compound 11c showed growth inhibitory effects that were stronger to TK-10 and MCF-7 cell lines and its inhibitory parameters (GI_50_, TGI and LC_50_) demonstrated total growth inhibition (TGI = 25.28, 21.87 µM/mL, respectively) compared to those presented by the rest of the group which affected only on one or two of the experimented cell lines. Compound **8 **showed an inhibitory effect against the growth of the TK-10 and MCF-7 cells, while the other derivatives **11a**,**b**,**e**–**h** showed no activities.

**Table 2:** Concentration (µM/mL) required to inhibit cell growth by 50% (GI_50_), to produce total growth inhibition (TGI) and to cause 50% net cell killing (LC_50_).

Product	Inhibition parameters	TK-10	MCF-7	UACC-62
**3c**	GI_50_	62.44 ± 3.58	80.66 ± 5.24	>100
	TGI	>100	>100	>100
	LC_50_	>100	>100	>100
**3d**	GI_50_	>100	95.48 ± 11.62	>100
	TGI	>100	>100	>100
	LC_50_	>100	>100	>100
**3f**	GI_50_	83.72 ± 9.73	>100	>100
	TGI	>100	>100	>100
	LC_50_	>100	>100	>100
**3i**	GI_50_	96.73 ± 11.27	81.29 ± 8.28	41.97 ± 4.97
	TGI	>100	>100	86.66 ± 7.14
	LC_50_	>100	>100	>100
**3j**	GI_50_	71.06 ± 4.87	63.87 ± 3.49	>100
	TGI	>100	>100	>100
	LC_50_	>100	>100	>100
**8**	GI_50_	72.08 ± 6.94	42.25 ± 4.11	>100
	TGI	>100	>100	>100
	LC_50_	>100	>100	>100
**11c**	GI_50_	16.39 ± 1.79	12.67 ± 1.08	43.78 ± 1.05
	TGI	25.28 ± 2.39	21.87 ± 2.10	>100
	LC_50_	38.99 ± 3.01	37.77 ± 3.91	>100

The range of doses assayed was 0.01–100 µM/mL; Results are mean ± SE (standard error of the mean) (*n* = 3); The GI_50_, TGI and LC_50 _for the other products are >100.

In conclusion, concerning the effect on the growth of human cancer cell lines, 11c was found to have the best inhibitory activity towards all the three cell lines, followed by **3i** that caused 50% cell growth inhibition on all the cancer cell lines. This activity could be attributed to heterocyclic fused rings system such as naphtha[1,8-*ef*]diazepines and pyrrolo[1,2-*a*]perimidine derivatives containing multiple centers with hydrogen accepting properties (COMe, C=O and nitrogen atoms), are essential for DNA intercalating activity and antitumor activity.

#### 2.2.2. Oxygen Radical Absorbance Capacity (ORAC) Assay

All the samples show weak antioxidant (ORAC) activity as scavengers. Compounds with antioxidant potential can directly interact with ROS, thereby reducing oxidative damage [41]. To investigate further the mechanism of action by which compounds **3**, **8** and **11** exert a protective effect under oxidative stress conditions, the potential radical-scavenging activity using the oxygen radical absorbance capacity (ORAC) chemical assay was evaluated.

The values of the area under the curve (AUC, Table 3) showed also that compounds **3**, **8** and **11** are much weaker scavengers than the reference compound Trolox, as the decline in fluorescence over time was significantly more rapid for all of the tested compounds than for Trolox (assayed at 8 μM concentration), indicating that they are not as effective as Trolox in scavenging peroxyl radicals generated by the thermal decomposition of 2,2'-azobis(2-amidinopropane) dihydrochloride (AAPH). Thus, because peroxy radicals are not effectively neutralized by the studied compounds, fluorescein is degraded, resulting in non-fluorescent products.

**Table 3:** ORAC values for antioxidant activity of compounds **3**, **8**, and **11**.

Comp. No.	ORAC Value	Comp. No.	ORAC Value
**3d**	0.61 ± 0.04	**11c**	0.86 ± 0.09
**3f**	0.74 ± 0.07	**11d**	0.43 ± 0.59
**3h**	0.54 ± 0.06	**11b**	0.51 ± 0.10
**3i**	0.97 ± 0.10	**11f**	0.60 ± 0.07
**3j**	0.68 ± 0.03	**11e**	0.62 ± 0.09
**3c**	0.81 ± 0.05	**11g**	0.42 ± 0.05
**3e**	0.65 ± 0.07	**11a**	0.53 ± 0.08
**8**	0.72 ± 0.06	**11h**	0.59 ± 0.06

Data expressed as mean ± SE (standard error of the mean) (*n* = 3), and ORAC values expressed as µM of Trolox/mg sample.

## 3. Experimental

### 3.1. General Information

All melting points were determined on an electrothermal Gallenkamp apparatus. Solvents were generally distilled and dried by standard literature procedures prior to use. The IR spectra were measured on a Pye-Unicam SP_300_ instrument in potassium bromide discs. The ^1^H-NMR spectra were recorded on a Varian Mercury VXR-300 MHz spectrometer (300 MHz for ^1^H-NMR and 75 MHz for ^13^C-NMR) and reported as the chemical shifts δ (ppm) downfield from tetramethylsilane (TMS) used as an internal standard. The mass spectra were recorded on a GCMS-Q1000-EX Shimadzu or GCMS 5988-A HP spectrometer; the ionizing voltage was 70 eV. Elemental analyses were carried out by the Microanalytical Center of Cairo University, Giza, Egypt. TLC was run on silica gel G coated plates and iodine vapour was used as visualizing agent.

### 3.2. General Procedure for the Reaction of Hydrazonoyl Chlorides with 1,8-Diaminonaphthalene

To a stirred solution of 1,8-diaminonaphthalene (**1**, 0.79 g, 5 mmol) and the appropriate hydrazonoyl chloride (5 mmol) in dioxane (40 mL), triethylamine (0.7 mL) was added dropwise and the mixture was then refluxed for 5 h. The precipitated triethylamine hydrochloride was filtered off and the filtrate was evaporated under reduced pressure. The residue was triturated with methanol. The solid product formed in each case was collected by filtration, washed with water, dried and recrystallized from a suitable solvent system as indicated in each example to afford the corresponding derivatives **3a**–**j** and **5b**,**d**,**f**.

*2-(4-Methoxyphenylazo)-3-methyl-1H-naphtho[1,8-ef][1,4]diazepine* (**3a**). Red solid (86%); mp 200–202 °C (EtOH); IR (KBr) ν_max_ 3394, 3286 (2NH) cm^−1^; ^1^H-NMR (DMSO-*d*_6_) δ 2.25 (s, 3H, CH_3_), 3.58 (s, 3H, OCH_3_), 6.65–7.92 (m, 10H, Ar-H), 9.98 (s, 1H, NH, D_2_O-exchangeable), 10.10 (s, 1H, NH, D_2_O-exchangeable); MS *m/z* (%) 331 (M^+^+1, 4), 330 (M^+^, 14), 327 (5), 140 (12), 123 (18), 122 (100), 107 (11), 77 (6). Anal. Calcd. for C_20_H_18_N_4_O (330.38): C, 72.71; H, 5.49; N, 16.96. Found: C, 72.49; H, 5.37; N, 16.75%.

*2-(4-Methylphenylazo)-3-methyl-1H-naphtho[1,8-ef][1,4]diazepine* (**3b**). Orange solid (88%); mp 240–242 °C (EtOH); IR (KBr) ν_max_ 3398, 3105 (2NH) cm^−1^; ^1^H-NMR (DMSO-*d*_6_) δ 2.28 (s, 3H, CH_3_), 2.35 (s, 3H, CH_3_), 7.13–7.55 (m, 10H, Ar-H), 10.63 (s, 1H, NH, D_2_O-exchangeable), 12.01 (s, 1H, NH, D_2_O-exchangeable); MS *m/z* (%) 315 (M^+^+1, 24), 314 (M^+^, 100), 313 (10), 297 (14), 209 (33), 167 (64), 140 (69), 106 (94), 91 (23), 77 (45). Anal. Calcd. for C_20_H_18_N_4_ (314.38): C, 76.41; H, 5.77; N, 17.82. Found: C, 76.26; H, 5.47; N, 17.61%.

*2-(3-Methylphenylazo)-3-methyl-1H-naphtho[1,8-ef][1,4]diazepine* (**3c**). Orange solid (78%); mp 150–152 °C (EtOH); IR (KBr) ν_max_ 3345, 3225 (2NH) cm^−1^; ^1^H-NMR (DMSO-*d*_6_) δ 2.25 (s, 3H, CH_3_), 2.32 (s, 3H, CH_3_), 6.85–7.37 (m, 10H, Ar-H), 9.85 (s, 1H, NH, D_2_O exchangeable), 10.20 (s, 1H, NH, D_2_O exchangeable); MS *m/z* (%), 315 (M^+^+1, 24), 314 (M^+^, 100), 313 (72), 297 (20), 280 (13), 208 (8), 167 (71), 147 (66), 140 (70), 107 (52), 91 (19), 77 (29). Anal. Calcd. for C_20_H_18_N_4_ (314.38): C, 76.41; H, 5.77; N, 17.82. Found: C, 76.24; H, 5.51; N, 17.64%.

*2-Phenylazo-3-methyl-1H-naphtho[1,8-ef][1,4]diazepine* (**3d**). Red solid (86%); mp 194–196 °C (EtOH); IR (KBr) ν_max_ 3215, 3154 (2NH) cm^−1^; ^1^H-NMR (DMSO-*d*_6_) δ 2.21 (s, 3H, CH_3_), 6.66–7.53 (m, 11H, Ar-H), 9.67 (s, 1H, NH, D_2_O-exchangeable), 9.90 (s, 1H, NH, D_2_O-exchangeable); MS *m/z* (%) 300 (M^+^, 45), 299 (35), 283 (17), 255 (21), 251 (20), 207 (41), 166 (100), 89 (21), 77 (21). Anal. Calcd. for C_19_H_16_N_4_ (300.36): C, 75.98; H, 5.37; N, 18.65. Found: C, 75.84; H, 5.21; N, 18.46%.

*2-(3-Chlorophenylazo)-3-methyl-1H-naphtho[1,8-ef][1,4]diazepine* (**3e**). Orange solid (75%); mp 230–232 °C (EtOH); IR (KBr) ν_max_ 3200, 3116 (2NH) cm^−1^; ^1^H-NMR (DMSO-*d*_6_) δ 2.31 (s, 3H, CH_3_), 7.01–7.89 (m, 10H, Ar-H), 10.20 (s, 1H, NH, D_2_O-exchangeable), 10.54 (s, 1H, NH, D_2_O-exchangeable); MS *m/z* (%) 336 (M^+^+2, 7), 335 (M^+^+1, 8), 334 (M^+^, 31), 208 (20), 166 (75), 140 (100), 111 (12), 90 (12), 75 (16). Anal. Calcd. for C_19_H_15_ClN_4_ (334.80): C, 68.16; H, 4.52; N, 16.73. Found: C, 68.05; H, 4.34; N, 16.41%.

*2-(4-Chlorophenylazo)-3-methyl-1H-naphtho[1,8-ef][1,4]diazepine* (**3f**). Deep-orange solid (83%); mp > 300 °C (EtOH/dioxane); IR (KBr) ν_max_ 3325, 3150 (2NH) cm^−1^; ^1^H-NMR (DMSO-*d*_6_) δ 2.27 (s, 3H, CH_3_), 6.76–7.58 (m, 10H, Ar-H), 9.91 (s, 1H, NH, D_2_O exchangeable), 10.01 (s, 1H, NH, D_2_O exchangeable); MS *m/z* (%) 336 (M^+^+2, 19), 335 (M^+^+1, 21), 334 (M^+^, 60), 208 (13), 166 (100), 139 (19), 126 (12), 113 (10), 111 (4), 63 (11). Anal. Calcd. For C_19_H_15_ClN_4_ (334.80): C, 68.16; H, 4.52; N, 16.73. Found: C, 68.05; H, 4.34; N, 16.41%.

*2-(3-Nitrophenylazo)-3-methyl-1H-naphtho[1,8-ef][1,4]diazepine* (**3g**). Green solid (77%); mp > 300 °C (EtOH/dioxane); IR (KBr) ν_max_ 3348, 3100 (2NH) cm^−1^; ^1^H-NMR (DMSO-*d*_6_) δ 2.31 (s, 3H, CH_3_), 6.89–8.07 (m, 10H, Ar-H), 8.31 (s, 1H, NH, D_2_O-exchangeable), 10.60 (s, 1H, NH, D_2_O exchangeable); MS *m/z* (%) 345 (M^+^, 68), 328 (4), 234 (8), 208 (19), 194 (15), 166 (100), 140 (93), 76 (6). Anal. Calcd. for C_19_H_15_N_5_O_2_ (345.35): C, 66.08; H, 4.38; N, 20.28. Found: C, 65.91; H, 4.11; N, 20.04%.

*2-(4-Nitrophenylazo)-3-methyl-1H-naphtho[1,8-ef][1,4]diazepine* (**3h**). Pale-brown solid (75%); mp 260–262°C (EtOH/dioxane); IR (KBr) ν_max_ 3382, 3290 (2NH) cm^−1^; ^1^H-NMR (DMSO-*d*_6_) δ 2.26 (s, 3H, CH_3_), 6.66–7.17 (m, 6H, Ar-H), 7.66 (d, *J* = 9 Hz, 2H, Ar-H), 8.17 (d, *J* = 9 Hz, 2H, Ar-H), 10.03 (s, 1H, NH, D_2_O-exchangeable), 10.43 (s, 1H, NH, D_2_O-exchangeable); MS *m/z* (%) 345 (M^+^, 69), 344 (54), 342 (43), 208 (29), 166 (100), 140 (57), 122 (20), 114 (46), 108 (46), 77 (26), 60 (63). Anal. Calcd. for C_19_H_15_N_5_O_2_ (345.35): C, 66.08; H, 4.38; N, 20.28. Found: C, 65.87; H, 4.20; N, 20.12%.

*2-(4-Acetylphenylazo)-3-methyl-1H-naphtho[1,8-ef][1,4]diazepine* (**3i**). Dark orange solid (78%); mp 280–282 °C (EtOH/dioxane); IR (KBr) ν_max_ 3394, 3286 (2NH), 1654 (C=O) cm^−1^; ^1^H-NMR (DMSO-*d*_6_) δ 2.25 (s, 3H, CH_3_), 2.51 (s, 3H, CH_3_), 6.65–7.20 (m, 6H, Ar-H), 7.59 (d, *J* = 9 Hz, 2H, Ar-H), 7.89 (d, *J* = 9 Hz, 2H, Ar-H), 9.98 (s, 1H, NH, D_2_O-exchangeable), 10.10 (s, 1H, NH, D_2_O-exchangeable); MS *m/z* (%) 342 (M^+^, 58), 341 (48), 208 (12), 166 (100), 163 (22), 140 (56), 139 (43), 120 (14), 91 (10). Anal. Calcd. for C_21_H_18_N_4_O (342.39): C, 73.67; H, 5.30; N, 16.36. Found: C, 73.52; H, 5.17; N, 16.15%.

*2-(4-Ethoxycarbonylphenylhydrazono)-3-methyl-1H-naphtho[1,8-ef][1,4] diazepine* (**3j**). Red solid (81%); mp 120–122 °C (EtOH); IR (KBr) ν_max_ 3200, 3150 (2NH), 1685 (CO) cm^−1^; ^1^H-NMR (DMSO-*d*_6_) δ 1.29 (t, *J* = 7 Hz, 3H, CH_3_), 2.35 (s, 3H, CH_3_), 4.27 (q, *J* = 7 Hz, 2H, CH_2_), 7.0–7.95 (m, 10H, Ar-H), 10.20 (s, 1H, NH, D_2_O-exchangeable), 10.65 (s, 1H, NH, D_2_O-exchangeable); MS *m/z* (%) 372 (M^+^, 47), 371 (20), 365 (60), 340 (60), 203 (13), 166 (100), 150 (40), 149 (80), 123 (80), 93 (100), 77 (73). Anal. Calcd. for C_22_H_20_N_4_O_2_ (372.42): C, 70.95; H, 5.41; N, 15.04. Found: C, 70.72; H, 5.19; N, 14.84%.

*2-(4-Methylphenylhydrazono)-1,4-dihydro-naphtho[1,8-ef][1,4]diazepine-3-one* (**5a**). Orange solid (80%); mp 100 °C (EtOH); IR (KBr) ν_max_ 3325, 3215, 3120 (3NH), 1658 (C=O) cm^−1^; ^1^H-NMR (DMSO-*d*_6_) δ 2.30 (s, 3H, CH_3_), 7.05–7.67 (m, 10H, Ar-H), 9.95 (s, 1H, NH, D_2_O-exchangeable), 10.15 (s, 1H, NH, D_2_O-exchangeable), 10.91 (s, 1H, NH, D_2_O-exchangeable); MS *m/z* (%) 316 (M^+^, 5), 315 (2), 225 (27), 197 (13), 134 (13), 106 (100), 105 (23), 91 (46), 89 (15), 77 (25). Anal. Calcd. for C_19_H_16_N_4_O (316.36): C, 72.13; H, 5.10; N, 17.71. Found: C, 71.96; H, 4.87; N, 17.50%.

*2-(4-Chlorophenylhydrazono)-1,4-dihydro-naphtho[1,8-ef][1,4]diazepine-3-one* (**5b**). Yellow solid (83%); mp 120–122 °C (EtOH); IR (KBr) ν_max_ 3315, 3204, 3210 (3NH), 1660 (C=O) cm^−1^; ^1^H-NMR (DMSO-*d*_6_) δ 6.94–7.85 (m, 10H, Ar-Hs), 9.87 (s, 1H, NH, D_2_O-exchangeable), 10.21 (s, 1H, NH, D_2_O exchangeable), 10.57 (s, 1H, NH, D_2_O exchangeable); MS *m/z* (%) 338 (M^+^+2, 10), 337 (M^+^+1, 1), 336 (M^+^, 35), 168 (100), 140 (25), 114 (11), 111 (1), 84 (19). Anal. Calcd. For C_18_H_13_ClN_4_O (336.77): C, 64.19; H, 3.89; N, 16.64. Found: C, 64.35; H, 3.66; N, 16.48%.

*Synthesis of 2-(2-hydroxybenzoylmethylene)-1,2-dihydro-perimidine* (**8**). A mixture of 1,8-diamino-naphthalene (**1**) (1.58, 10 mmol) and 4-hydroxycoumarin (**6**, 1.62 g, 10 mmol) in ethanol (20 mL) was heated to reflux for 5 h, then cooled. The solid formed was collected by filtration and washed with ethanol, then crystallized from a dioxane/ethanol mixture to give compound **8** as golden yellow crystals, (89%), mp 300 °C, IR (KBr) ν_max_ 3490, 3220, 3116 (2NH, OH), 1647 (CO) cm^−1^; ^1^H-NMR (DMSO-*d*_6_) 5.62 (s, 1H, CH), 6.63–7.572 (m, 10H, Ar-H), 10.91 (s, 1H, NH), 12.63 (s, 1H, OH), 13.78 (s, 1H, NH); ^13^C-NMR (DMSO-*d*_6_): 77.15 (=CH), 106.0, 116.37, 117.76, 118.41, 119.11, 119.52, 120.26, 126.81, 128.34, 132.99, 134.13, 153.50, 161.22 (Ar-C), 186.22 (C=O). MS *m/z* (%) 302 (M^+^, 67), 301 (47), 182 (100), 168 (20), 127 (19), 121 (45), 114 (12), 93 (12). Anal. Calcd. For C_19_H_14_N_2_O_2_ (302.33) C, 75.48; H, 4.67; N, 9.27. Found: C, 75.25; H, 4.41; N, 9.46%.

Reaction of compound **8** with hydrazonoyl chlorides **4a**–**h**. To a mixture of compound **8** (5 mmol) and the appropriate hydrazonoyl chloride **4a**–**h** (5 mmol of each) in dioxane (30 mL) was added triethylamine (0.7 mL) and the mixture was heated to reflux for 10 h, then cooled. The solid produced was collected by filtration and crystallized from the appropriate solvent to give the corresponding compounds **11a**–**h**.

*8-(2-Hydroxybenzoyl)-7H-9-(4-methylphenylhydrazono)-pyrrolo[1,2-a] perimidin-10-one* (**11a**). Orange solid, (82%); mp > 320 °С (ethanol/dioxane), IR (KBr) ν_max_ 3200–3100 (2NH, OH), 1690, 1639 (2C=O) cm^−1^, ^1^H-NMR (DMSO-*d*_6_) δ: 2.19 (s, 3H, CH_3_), 6.73–7.67 (m, 14H, Ar-H), 9.89 (s, 1H, NH), 12.04 (s, 1H, OH), 13.80 (s, 1H, NH); MS *m/z* (%) 460 (M^+^, 13), 459 (11), 458 (18), 340 (17), 339 (44), 338 (52), 283 (9), 248 (7), 221 (6), 167 (15), 166 (27), 152 (21), 127 (26), 121 (43), 106 (40), 91 (54), 84 (98), 84 (64), 77 (37), 55 (100). Anal. Calcd. for C_28_H_20_N_4_O_3_ (460.48): C, 73.03; H, 4.38; N, 12.17. Found: C, 73.31; H, 4.17; N, 11.97%.

*8-(2-Hydroxybenzoyl)-7H-9-(3-methylphenylhydrazono)-pyrrolo[1,2-a] perimidin-10-one* (**11b**). Orange solid, (79%); mp 288–290 °С (ethanol/dioxane), IR (KBr) ν_max_ 3236, 3100 (2NH, OH), 1689, 1647 (2C=O) cm^−1^, ^1^H-NMR (DMSO-*d*_6_) δ: 2.20 (s, 3H, CH_3_), 6.61–8.50 (m, 14H, Ar-H), 9.85 (s, 1H, NH), 11.70 (s, 1H, OH), 12.03 (s, 1H, NH); MS *m/z* (%) 460 (M^+^, 13), 459 (15), 458 (15), 340 (15), 339 (38), 338 (100), 337 (35), 254 (24), 178 (21), 169 (29), 138 (29), 127 (29), 124 (18), 93 (27), 92 (63), 91 (38), 84 (18), 83 (21), 77 (59), 76 (32). Anal. Calcd. for C_28_H_20_N_4_O_3_ (460.48): C, 73.03; H, 4.38; N, 12.17. Found: C, 73.25; H, 4.09; N, 11.91%.

*8-(2-Hydroxybenzoyl)-7H-9-phenylhydrazono-pyrrolo[1,2-a]perimidin-10-one* (**11c**). Yellow solid, (85%); mp: 275–277 °С (ethanol/dioxane), IR (KBr) ν_max_ 3429, 3249 (2NH, OH), 1689, 1643 (2C=O) cm^−1^, ^1^H-NMR (DMSO-*d*_6_) δ: 6.77–8.47 (m, 15H, Ar-H), 10.0 (s, 1H, NH), 11.70 (s, 1H, OH), 12.02 (s, 1H, NH); MS *m/z* (%) 446 (M^+^, 27), 445 (23), 444 (23), 175(18), 140 (18), 120 (32), 95 (27), 90 (32), 82 (41), 77 (32), 64 (100). Anal. Calcd. for C_27_H_18_N_4_O_3_ (446.46): C, 72.64; H, 4.06; N, 12.55. Found: C, 72.51; H, 4.13; N, 12.29%.

*8-(2-Hydroxybenzoyl)-7H-9-(4-chlorophenylhydrazono)-pyrrolo[1,2-a] perimidin-10-one* (**11d**). Orange solid, (83%); mp 308–310 °С (ethanol/dioxane), IR (KBr) ν_max_ 3430–3247 (br. 2NH, OH), 1688, 1644 (2C=O) cm^−1^, ^1^H-NMR (DMSO-*d*_6_) δ: 6.70–8.39 (m, 14H, Ar-H), 9.80 (s, 1H, NH), 9.98 (s, 1H, OH), 11.94 (s, 1H, NH); MS *m/z* (%) 480 (M^+^, 11), 479 (15), 339 (46), 338 (100), 337 (21), 254 (14), 178 (29), 169 (16), 138 (29), 127 (29), 111 (43), 92 (63), 91 (24), 76 (31). Anal. Calcd. for C_27_H_17_ClN_4_O_3_ (480.90): C, 67.43; H, 3.56; N, 11.65. Found: C, 67.20; H, 3.32; N, 11.45%.

*8-(2-Hydroxybenzoyl)-7H-9-(4-bromophenylhydrazono)-pyrrolo[1,2-a] perimidin-10-one* (**11e**). Dark red solid, (84%); mp 278–280 °С (ethanol/dioxane), IR (KBr) ν_max_ 3413, 3200, 3150 (2NH, OH), 1689, 1647 (2C=O) cm^−1^, ^1^H-NMR (DMSO-*d*_6_) δ: 6.70–8.44 (m, 14H, Ar-H), 9.61 (s, 1H, NH), 9.97 (s, 1H, OH), 11.94 (s, 1H, NH); MS *m/z* (%) 526 (M^+^+1, 20), 525 (M^+^, 20), 524 (25), 523 (41), 505 (25), 340 (29), 339 (94), 338 (100), 254 (29), 219 (16), 179 (22), 173 (31), 166 (67), 155 (37), 138 (29), 121 (69), 104 (27), 93 (27), 92 (39), 91 (37), 77 (39), 76 (43), 75 (43). Anal. Calcd. for C_27_H_17_BrN_4_O_3_ (525.35): C, 61.73; H, 3.26; N, 10.66. Found: C, 61.54; H, 3.05; N, 10.47%.

*8-(2-Hydroxybenzoyl)-7H-9-(3-nitrophenylhydrazono)-pyrrolo[1,2-a]perimidin-10-one* (**11f**). Orange solid, (76%); mp > 320 °С (ethanol/dioxane), IR (KBr) ν_max_ 3433, 3235 (2NH, OH), 1687, 1647 (2C=O) cm^−1^, ^1^H-NMR (DMSO-*d*_6_) δ: 6.86–8.43 (m, 14H, Ar-H), 10.05 (s, 1H, NH), 12.20 (s, 1H, OH), 12.44 (s, 1H, NH); MS *m/z* (%) 491 (M^+^, 20), 490 (15), 489 (29), 473 (29), 340 (21), 339 (65), 338 (100), 337 (46), 253 (25), 184 (15), 166 (32), 152 (26), 121 (44), 93 (22), 76 (47). Anal. Calcd. for C_27_H_17_N_5_O_5_ (491.45): C, 65.99; H, 3.49; N, 14.25. Found: C, 65.75; H, 3.21; N, 14.08%.

8-(2-Hydroxybenzoyl)-7H-9-(4-nitrophenylhydrazono)-*pyrrolo[1,2-a]perimidin*-10-one (**11g**). Red solid, (81%); mp 310–313 °С (dioxane), IR (KBr) ν_max_ 3400, 3350, 3220 (2NH, OH), 1693, 1647 (2C=O) cm^−1^, ^1^H-NMR (DMSO-*d*_6_) δ: 6.90–7.92 (m, 14H, Ar-H), 9.79 (s, 1H, NH), 11.82 (s, 1H, OH), 12.17 (s, 1H, NH); MS *m/z* (%) 491 (M^+^, 5), 490 (12), 489 (28), 473 (62), 338 (100), 253 (22), 179 (11), 166 (31), 152 (19), 121 (29), 104 (18), 93 (22), 76 (57). Anal. Calcd. for C_27_H_17_N_5_O_5_ (491.45): C, 65.99; H, 3.49; N, 14.25. Found: C, 65.69; H, 3.30; N, 14.45%.

*8-(2-Hydroxybenzoyl)-7H-9-(4-acetylphenylhydrazono)-pyrrolo[1,2-a]perimidin-10-one* (**11h**). Orange solid, (82%); mp 298–300 °С (dioxane), IR (KBr) ν_max_ 3350, 3240, 3100 (2NH, OH), 1700, 1689, 1647 (3C=O) cm^−1^, ^1^H-NMR (DMSO-*d*_6_) δ: 2.47 (s, 3H, CH_3_), 6.85–8.49 (m, 14H, Ar-H), 9.84 (s, 1H, NH), 11.80 (s, 1H, OH), 12.13 (s, 1H, NH); MS *m/z* (%) 488 (M^+^, 46), 338 (55), 326 (55), 153 (46), 137 (64), 135 (73), 121 (82), 104 (64), 92 (55), 84 (64), 77 (91), 76 (73), 60 (100). Anal. Calcd. for C_29_H_20_N_4_O_4_ (488.49): C, 71.30; H, 4.13; N, 11.47. Found: C, 71.14; H, 4.0; N, 11.19%.

### 3.3. ORAC Assay

Scavenging activity was measured by using an ORAC assay [42]. Dilutions of the samples **3c**–**f**,**h**–**j**, **8**, and **11a**–**h** and Trolox (as antioxidant reference compound) were incubated for 10 min at 37 °C in fluorescein (70 nM final concentration). After this incubation period, 2,2''-azobis(2-amidinopropane) dihydrochloride (12 mM final concentration) was added to the mixture. Fluorescence was read every 56 s for 98 min using a FLUOstar Optima (BMG Labtech, Headquarters, Germany) fluorometer. Area under curve values were calculated for each sample and compared with the AUC corresponding to Trolox. Results are expressed as µM of Trolox equivalents/mg of sample.

### 3.4. Human Cell Lines for Cytotoxicity Assays

The human renal denocarcinoma (TK-10), the human breast adenocarcinoma (MCF-7) and the human melanoma (UACC-62) Cell lines were used in these experiments. The human tumour cytotoxicities were determined following protocols established by the NCI [43]. TK-10, MCF-7 and UACC-62 cell lines were cultured in RPMI 1640 medium (Bio Whittaker, Basel, Switzerland) containing 20% foetal calf serum (FCS), 2 mM L-glutamine, 100 µg/mL penicillin and 100 µg/mL streptomycin. All cell lines were maintained at 37 °C in a 5% CO_2_ atmosphere with 95% humidity. Maintenance cultures were passaged weekly, and the culture medium was changed twice a week.

### 3.5. Testing Procedure and Data Processing

The sulforhodamine B (SRB) assay is used for cell density determination, based on the measurement of cellular protein content. The method described here has been optimized for the toxicity screening of compounds to adherent cells in a 96-well format. Viable cells were counted using a Coulter counter and diluted with medium to give final densities of 15 × 10^4^, 5 × 10^4^ and 100 × 10^4^ cells/mL for TK-10, MCF-7and UACC-62, respectively. After 24 h, the cells were treated with the serial concentrations of extracts. One hundred microlitres per well of each concentration was added to the plates to obtain final concentration of 10^−4^, 10^−5^, 10^−6^, 10^−7^ and 10^−8 ^M. The final volume in each well was 200 µg/mL. The plates were incubated for 48 h.

### 3.6. Sulphorhodamine B Method

After an incubation period for 48 h, cell monolayers are fixed with 50 µL of cold 50% (wt/vol) trichloroacetic acid (TAC) and stained for 60 min at 4 °C, after which the excess dye (SRB solution 0.4% w/v in 1% acetic acid) is removed after 30 min incubated, by washing repeatedly with 1% (vol/vol) acetic acid. The protein-bound dye is dissolved in 10 mM Tris base solution for OD determination at 492 nm using a microplate reader. At the end, GI_50_ values (concentrations required to inhibit cell growth by 50%), TGI (concentration resulting in total growth inhibition) and LC_50_ (concentration causing 50% of net cell killing) were calculated according to the previously described protocols [43]. Two or three experiments were carried out for each extract or compound. The data are given as the mean of three different assays ± SE.

## 4. Conclusions

Highly efficient and simple methods were described for the preparation of two series of naphtho[1,8-*ef*][1,4]diazepines and pyrrolo[1,2-*a*]perimidines from 1,8-diaminonaphthalene and hydrazonoyl chlorides. The mechanism of formation of such products was discussed. The newly synthesized compounds were examined for *in vitro* activity against several human cancer cell lines, including TK-10, MCF-7 and UACC-62. Compound **11c** was found to have the best inhibitory activity towards all the three cell lines, followed by **3i** that caused inhibition of 50% of cell growth on all the cancer cell lines.
